# Electrodeposition of Polyaniline on Tantalum: Redox Behavior, Morphology and Capacitive Properties

**DOI:** 10.3390/molecules28217286

**Published:** 2023-10-26

**Authors:** Chrysanthi Gkili, Konstantinos Deligiannakis, Eirini Lappa, Chrysanthi Papoulia, Dimitra Sazou

**Affiliations:** 1 Department of Chemistry, Aristotle University of Thessaloniki, 54124 Thessaloniki, Greece; chrysagkili@gmail.com (C.G.); konstantinosdel32@gmail.com (K.D.); eirlap86@gmail.com (E.L.); 2 Department of Physics, Aristotle University of Thessaloniki, 54124 Thessaloniki, Greece; cpapouli@physics.auth.gr

**Keywords:** tantalum oxide, polyaniline nanospheres/nanorods, oxide–polyaniline composite, cathodic peaks, cyclic voltammetry, specific capacitance

## Abstract

Polyaniline (PANI) is among the most widely studied conducting polymers due to its potential technological applications in various fields. Recently, PANI-based hybrid materials have played an important role in the development of energy storage and conversion systems. The aim of the present work is the investigation of the simultaneous electrochemical growth of PANI and Ta_2_O_5_ on the Ta substrate and the characterization of the morphology, redox behavior and pseudocapacitive properties of the resulting micro- or nanostructured composite thin films. A well-adherent conductive Ta_2_O_5_-PANI composite film was first formed using cyclic voltammetry on Ta that facilitates the on-top electrodeposition of single PANI via an autocatalytic mechanism. The electrochemical characterization of the Ta|Ta_2_O_5_-PANI|PANI electrodes reveals unique redox properties of PANI not shown previously upon using PANI electrodeposition on Ta. Scanning electron microscopy shows that the morphology of the electrodeposited films comprises nano- or microspheres that may develop into nano- or microrods when the polymerization proceeds. Preliminary evaluation of the capacitive properties of the Ta|Ta_2_O_5_-PANI|PANI electrode shows adequately high specific capacitance values as high as 1130 F g^−1^ (at 9.2 mA cm^−2^), depending on the electrochemical parameters, as well as adequate stability (~80% retention after 100 cycles), indicating their potential application as energy storage devices.

## 1. Introduction

Conducting polymers (CPs) have attracted considerable attention as organic polymers due to their potential applications stemming from their unique physicochemical properties, such as their electrochemical activity, nanostructured morphology and closely associated optical, electrical and electronic properties [1]. Among CPs, polyaniline (PANI) is widely studied not only because of its easy, low-cost chemical or electrochemical preparation and adequate stability but also due to its unique doping/de-doping properties and a variety of recently revealed nanostructures that led to more advanced applications [2,3,4,5,6,7].

Nowadays, PANI is considered as a promising material for energy storage devices such as rechargeable batteries [8], solar-cell devices [9] and electrochemical supercapacitors [10]. The main materials that have been investigated for a supercapacitor electrode [11] are carbon [12], metal oxide [13,14] and polymers such as CPs [15]. Essential factors controlling the capacitive properties of PANI include its electrochemical behavior, molecular structure, and morphology, which are primarily determined via the polymerization method, the nature of the used dopant and the substrate. The electrodeposition of PANI onto different metal substrates, ranging from less active (Pt, Au, carbons) to active materials (e.g., Fe, Cu, stainless steel), has been widely studied [16,17,18]. It was observed that, depending on the substrate, as well as the electrochemical technique and the electrolyte, used, the electrochemical synthesis leads to the direct formation of a variety of nanostructured thin films ready to be used as a part of a device [19]. A limited number of studies have been reported on valve metal substrates such as Ti [20,21,22,23], Nb [24,25] and Ta [26,27], where the PANI electrodeposition process exhibits substantial differences due to the rapid formation of a high-resistance oxide layer on the metal’s surface. Compared to conventional substrates (Pt, Au or carbons), a lower polymerization rate is expected on valve metals, which, in the case of Ti, seems to depend on the electronic properties of TiO_2_ and increases upon increasing the level of oxygen deficiency [23].

The simultaneous nucleation and growth of PANI and metal oxide on valve metals affects both the redox behavior and morphology of the electrodeposited thin film, depending on the polymerization conditions. Indeed, in the case of Ti and Nb, an additional reduction peak is observed in the cyclic voltammogram (CV) of the PANI-modified electrodes at potential values close to the flat-band potential (*E*_FB_) of TiO_2_ and Nb_2_O_5_, respectively. The origin of this peak is still disputable. In the case of Ti, the additional peak was correlated either to the presence of an intermediate species, which might react to form phenazine rings [21], or the n-type semiconducting properties of TiO_2_ [23]. In the case of Nb, the additional cathodic peak was tentatively assigned to degradation products trapped inside the film or the reduction of Nb_2_O_5_ [24]. Though several open questions regarding the electrochemical behavior of PANI formed on valve metal substrates still remain, a previous study on the growth of PANI on Ti via cyclic voltammetry [23] indicated that the electrochemical polymerization of aniline (ANI) on Ti, as well as on other valve metals, may result in the formation of a composite oxide-PANI film (a dash is used throughout the text to represent the PANI-modified oxide film comprising a composite material), which was found to possess a higher conductivity compared to the single oxide, hence facilitating the electrochemical synthesis of PANI. Thus, on top of this conductive composite film, pure PANI grows readily at relatively high rates depending on the oxide electronic properties. These PANI-based composite films formed directly on a stable substrate are considered as interesting promising multifunctional systems in many applications.

In this study, the direct electrodeposition of PANI on Ta|Ta_2_O_5_ electrodes (a vertical line is used throughout the text to represent interfaces between different materials) is carried out from 0.2 M ANI-containing 1 M H_2_SO_4_ aqueous solution, aiming to form a composite Ta_2_O_5_-PANI thin film of a relatively high conductivity, where the growth of PANI and perhaps of other CPs on top of it is expected to be enhanced. The goal of this study is two-fold. Firstly, we aim to explore the redox behavior of the Ta|Ta_2_O_5_-PANI|PANI electrodes and gain insights into the polymerization mechanism associated with the appearance of additional cathodic peaks and, secondly, we aim to investigate the electrochemical properties and morphology of the resulting polymer films in terms of their potential use in energy storage systems. By extending the lower-potential limit up to −1.2 V_SCE_, additional cathodic peaks were revealed in the CV of the Ta|Ta_2_O_5_-PANI|PANI electrodes. Notably, they are reported here for the first time in the same manner as depicted in previous investigations regarding the electrodeposition of PANI on Ta [21,22]: a lower limit equal to −0.2 V was used. The peak located at less negative potentials bears a great resemblance to an additional cathodic peak reported in the literature for the electrodeposition of PANI on Ti [21,23] and Nb [24], whereas the other peak located at more negative potentials was never reported for either Ti or Nb. The involvement of the tantalum oxide in the polymerization process becomes evident, leading to the formation of a two-layer film comprising an inner hybrid Ta_2_O_5_-PANI layer and an outer PANI layer. The morphology of both the composite Ta_2_O_5_-PANI film and pure PANI film was investigated using scanning electron microscopy (SEM), whereas the electrochemical capacitance performance of the Ta|Ta_2_O_5_-PANI|PANI electrodes was evaluated in 1 M H_2_SO_4_ aqueous solution. These preliminary results indicate a relatively high specific capacitance and a high-rate capability for the PANI-based tantalum electrode.

## 2. Results

### 2.1. Cyclic Voltammetric Deposition of PANI on the Ta|Ta_2_O_5_ Electrode

**Figure 1:**
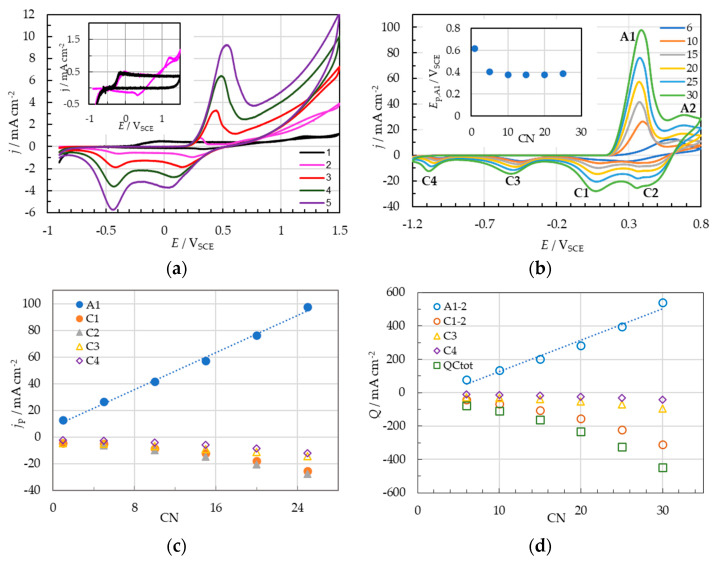
CVs representing current density–potential (*j*–*E*) curves of the Ta electrode in 1 M H_2_SO_4_ containing 0.2 M ANI at a scan rate of *υ* = 50 mV s^−1^ within the potential region: (**a**) −0.9–1.5 V_SCE_ (5 potential cycles) and (**b**) −1.2–0.8 V_SCE_ (25 potential cycles). Effect of the cycle number CN on (**c**) the peak current density *j*_p_ and (**d**) the charge density, with *Q* included under the various peaks that emerged in CVs during the polymerization process. The inset in (**a**) shows the magnification of the 1st potential cycle (CN = 1, black line) in comparison to the electrochemical behavior of Ta in an ANI-free 1 M H_2_SO_4_ solution (pink line). The inset in (**b**) shows the shift of *E*_p,A1_ toward lower values after restricting the upper anodic potential during the polymerization in the potential range of −1.2–0.8 V_SCE_. The dash line in (**c**) and (**d**) represents a regression line.

The electrochemical deposition of PANI on the Ta|Ta_2_O_5_ electrode was carried out from 1 M H_2_SO_4_ containing 0.2 M ANI using cyclic voltammetry at a potential scan rate of *υ* = 50 mV s^−1^, as illustrated in Figure 1. Τhe initiation step of the ANI polymerization consisted of five sequential potential cycles (Figure 1a) scanned within the potential region of −0.9–1.5 V_SCE_. Subsequently, 25 potential cycles were scanned successively at the same *υ* within the region of −1.2–0.8 V_SCE_ (Figure 1b). The lower and upper potential limits of cyclic voltammograms (CVs) traced after the initiation step were chosen as follows:

- We aimed to prevent the formation of PANI hydrolysis products, which might occur due to possible overoxidation of PANI at relatively high potential values (>0.8 V_SCE_).
- We aimed to detect any possible cathodic peaks that could emerge during the successive potential sweeps.

The first cycle traced with and without ANI (inset of Figure 1a) shows an increase in the current at ~−0.25 V_SCE_, reaching a maximum value at −0.11 V_SCE_ during the forward scan. Then, a plateau is formed at −0.025 V_SCE_. This is the characteristic electrochemical behavior of Ta in 1 M H_2_SO_4_, where electrodissolution and then passivation occurs [28], leading to the formation of an oxide layer [29] in accordance with the following reaction:

2Ta + 5H_2_O ⟶ Ta_2_O_5_ + 10 H^+^ + 10e^−^ (1)

In the presence of ANI, the electrochemical oxidation of ANI is distinguished at ~1 V_SCE_, where the current again starts to increase, and a new peak appears at *E*~1.2 V_SCE_. It seems that this reaction occurs on the passive Ta electrode during both the forward and backward potential scans. The increase in the current observed beyond the second narrow plateau region at *E* > 1.3 V_SCE_, along with the double trace crossing of the anodic current shown in the reverse backward scan of the first cycle, is indicative of slow-rate follow-up reactions taking place in the interfacial diffusive layer adjacent to the Ta electrode. These may involve an autocatalytic generation and perhaps a subsequent coupling of cation radicals and the formation of oligomers as it is manifested via the reduction peak observed at *E*_p_~0.33 V_SCE_ during the reverse backward potential scan.

During the second and subsequent cycles, a redox peak is established in the region of −0.2 and 0.5 V_SCE_, whereas an additional cathodic peak appears around −0.4 V_SCE_, indicating the formation of PANI, for which redox processes occur at lower potentials than those expected for ANI or its oligomers. After the 1st potential cycle, the current increases with the cycle number (CN), indicating an increase in the amount of PANI electrodeposited on the Ta|Ta_2_O_5_ substrate. As can be seen in Figure 1b, the current continues to increase with CN during the next 25 cycles scanned in the potential window of −1.2–0.8 V_SCE_. This behavior is in agreement with an autocatalytic mechanism suggested in the literature for the ANI polymerization [30].

Eventually, two anodic peaks designated as A1 and A2 in Figure 1b are clearly distinguished in the potential region of 0.35–0.5 V_SCE_, with their counterpart cathodic peaks C1 and C2, respectively, found in the region of 0–0.33 V_SCE_. The two redox pairs, namely A1/C1 and A2/C2, presumably correspond to the two-step reversible transitions of PANI from leucoemeraldine (LE) to emeraldine salt (ES) and ES to pernigraniline (PN), respectively [30]. Some smaller peaks that frequently accompany the main redox peaks due to the presence of electroactive degradation products cannot be clearly distinguished between A1 and A2. These peaks, also called “middle” peaks, may merge with the main redox peaks, resulting in broad CVs. Nevertheless, distinct “middle” peaks are facilitated at much lower concentrations of ANI [21] than the concentration of 0.2 M used in the present study.

As can be seen in Figure 1b, two additional cathodic peaks C3 and C4 appear at more negative potentials, namely *E*_p,C3_~−0.45 and *E*_p,C4_~−1.1 V_SCE_, which are not observed during the electrodeposition of PANI on typical substrates such as Pt, Au, glassy carbon or stainless steel. Peak C4 was not previously reported in the literature, whereas peak C3 was reported in the case of Ti [21,23] and Nb [24]. However, as was already mentioned, an explicit assignment of peak C3 has not yet been reported. The possible origins of peaks C3 and C4 will be discussed below.

The peak current density *j*_p_ corresponding to anodic and cathodic processes linearly increases with increasing CN during the polymerization process in both potential regions. Figure 1c shows the dependence of *j*_p_ on CN for 25 cycles scanned within the region of −1.2–0.8 V_SCE_ after the initiation step of 5 cycles. The slope d*j*_p,A_/dCN is indicative of the ANI polymerization rate, since CN can be considered as a measure of the polymerization time. It was found that d*j*_p,A_/dCN = 3.5 mA cm^−2^ per cycle number.

Moreover, the charge density *Q*_A_ included under the anodic CVs (peaks A1 and A2, designated as A1–2), as well as the charge density under the cathodic peaks (peaks C1 and C2, designated as C1–2, as well as C3 and C4), was evaluated via the integration of the area under the corresponding CVs. Figure 1d shows that the charge densities of anodic and cathodic peaks vary linearly with the CN. It is noteworthy that the total anodic charge density *Q*_A_ is almost equal to the total cathodic charge density *Q*_Ctot_. The slope d*Q*_A_/dCN of the *Q*_A_ = f(CN) plot is indicative of the electrodeposition rate and provides a measure of the increase in the PANI film thickness. It was found that d*Q*_A_/dCN = 18.8 mQ cm^−2^ per cycle number. The *j*_p,A_ = f(CN) and *Q*_A_ = f(CN) plots showing linearity can be used to regulate the amount of PANI deposited on Ta and hence the thickness of the PANI film, which, under potentiodynamic polymerization conditions used for total 30 cycles, was estimated to fluctuate between approximately 18 and 20 μm.

### 2.2. Electrochemical Response of the Ta|Ta_2_O_5_-PANI|PANI Electrode

**Figure 2:**
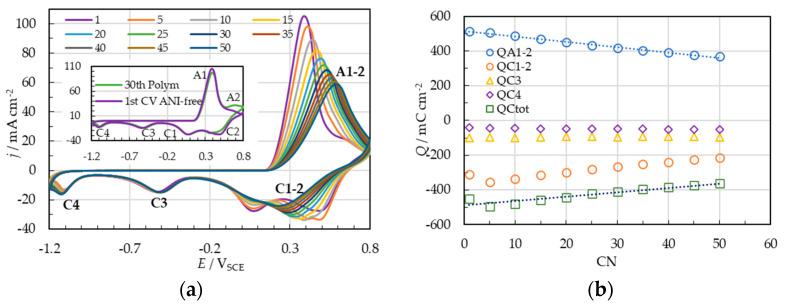
(**a**) The electrochemical response of the Ta|Ta_2_O_5_-PANI|PANI electrode in the ANI-free 1 M H_2_SO_4_ aqueous solution illustrating (**a**) consecutive CVs within the potential region of −1.2–0.8 V_SCE_ traced at *υ* = 50 mV s^−1^. (**b**) The effect of successive sweeps on the charge density of the anodic and cathodic peaks representing the redox behavior of the Ta|Ta_2_O_5_-PANI|PANI electrode. The inset in panel (**a**) shows the last CV obtained at the 30th cycle in the polymerization solution (Figure 1b) in comparison to the first CV traced for the Ta|Ta_2_O_5_-PANI|PANI electrode in the ANI-free 1 M H_2_SO_4_ solution. The dash line in (**b**) represents a regression line.

CVs in the inset of Figure 2a show the electrochemical response of the prepared Ta|Ta_2_O_5_-PANI|PANI electrode in an ANI-free 1 M H_2_SO_4_ solution in comparison to the CV corresponding to the last potential cycle, traced during the polymerization process within the potential region −1.2–0.8 V_SCE_ (Figure 1b). It seems that these two CVs are almost identical, indicating the stability of the electrode in the AN-free solution. A difference is that the anodic peak A2 becomes ill-defined in the ANI-free solution, while its counterpart cathodic peak is not extensively affected. Redox couples observed at peak potentials *E*_p,A1_=0.4 V_SCE_, *E*_p,C1_=0.1 V_SCE_ and *E*_p,A2_=0.68, *E*_p,C2_=0.45 V_SCE_ are assigned to the expected PANI interconversions, namely between LE and ES and ES and PN, respectively, as also observed in the CVs traced during the polymerization process.

The stability of the Ta|Ta_2_O_5_-PANI|PANI electrode was further assessed by examining the electrochemical behavior of the Ta|Ta_2_O_5_-PANI|PANI electrode in the ANI-free 1 M H_2_SO_4_ solution toward consecutive potential cycling at *υ* = 50 mV s^−1^. As seen in Figure 2a, both the distinct anodic peaks A1 and A2 and their cathodic counterparts C1 and C2 shift to higher and lower potentials, respectively, during consecutive potential cycling, displaying a tendency to merge into a single anodic and cathodic peak, respectively. After approximately 50 cycles, an almost steady-state CV is established. In particular, the peak potential *E*_p,A1–2_~0.6 V_SCE_ is shifted to more positive values with respect to *E*_p,A1_ by almost 0.2 V _SCE_ and to less positive values by almost 0.05 V_SCE_ with respect to *E*_p,A2_. Correspondingly, the peak potential *E*_p,C1–2_~0.2 V_SCE_ is shifted to more positive values with respect to *E*_p,C1_ by almost 0.15 V_SCE_ and to less positive values by almost 0.3 V_SCE_ with respect to *E*_p,C2_. There is also a decrease in *j*_p_, which, however, tends toward a steady-state value upon cycling.

Due to changes in the initial shape of the CV, the charge density included under the broad anodic A1–2 and cathodic C1–2 peaks is perhaps a better indicator than *j*_p_ of the stability of the Ta|Ta_2_O_5_-PANI|PANI electrode. Figure 2b shows the effect of the cycle number on the charge densities *Q*_A_ and *Q*_C_ included under the broad anodic A1–2 and cathodic C1–2 peaks, as well as under the cathodic peaks C3 and C4. Both charge densities *Q*_C3_ and *Q*_C4_, corresponding to peaks C3 and C4, respectively, remain almost constant, indicating that the prepared electrodes are adequately stable, whereas the total anodic and cathodic charge density is reduced by approximately 25%. However, this diminution cannot be seen as a loss given that the observed changes in the CV and the wide peak separation are characteristic of solid-state transformations that seem able to occur considering the redox mixed phases due to the bilayer structure of the Ta|Ta_2_O_5_-PANI|PANI electrode, comprising an inner Ta_2_O_5_-PANI composite layer and an outer single PANI layer [31,32,33].

**Figure 3:**
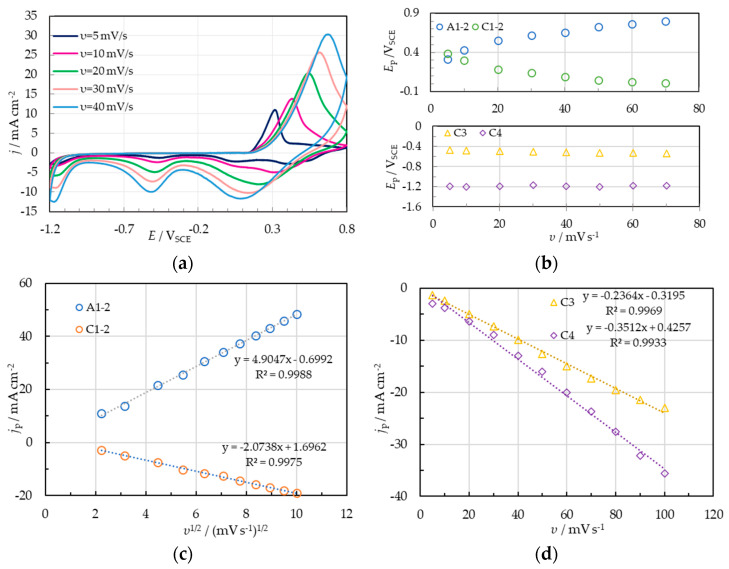
(**a**) The electrochemical response of the Ta|Ta_2_O_5_–PANI|PANI electrodes comprising CVs at different *υ* values in an ANI-free 1 M H_2_SO_4_ solution in the potential region between −1.2 and 0.8 V_SCE_. (**b**) The effect of *υ* on the peak potential *E*_p_ of the anodic A1–2 and cathodic C1–2 peaks. (**c**) Linear dependence of the peak current density *j*_p_ of the anodic A1–2 and cathodic C1–2 peaks on *υ*^1/2^. (**d**) Linear dependence of *j*_p_ of the cathodic peaks C3 and C4 on *υ*.

Figure 3a illustrates the effect of the potential scan rate *υ* on the redox peaks observed in the CV of the Ta|Ta_2_O_5_-PANI|PANI electrode after 50 potential cycles in the region of −1.2–0.8 V_SCE_. As seen in Figure 3b, the peak separation between the broad anodic A1–2 and cathodic C1–2 peaks gradually increases upon increasing *υ* from 5 to 50 mV s^−1^, reaching a value of ~0.7 V. The peak potential for both cathodic peaks C3 and C4 remains almost constant.

Figure 3c shows that the peak current densities *j*_p_ for peaks A1–2 and C1–2 linearly increase with the square root of the potential scan rate υ^1/2^. This behavior points to a mass transport-limited process that could be due to the outward and inward diffusion of protons and/or HSO_4^−^_ during the oxidation and reduction of PANI, non-excluding other associated processes such as swelling/deswelling and solid-state transformations. On the contrary, the *j*_p_ values of both cathodic peaks C3 and C4 linearly increase with *υ* (Figure 3d), as expected for surface-bound electroactive species.

**Figure 4:**
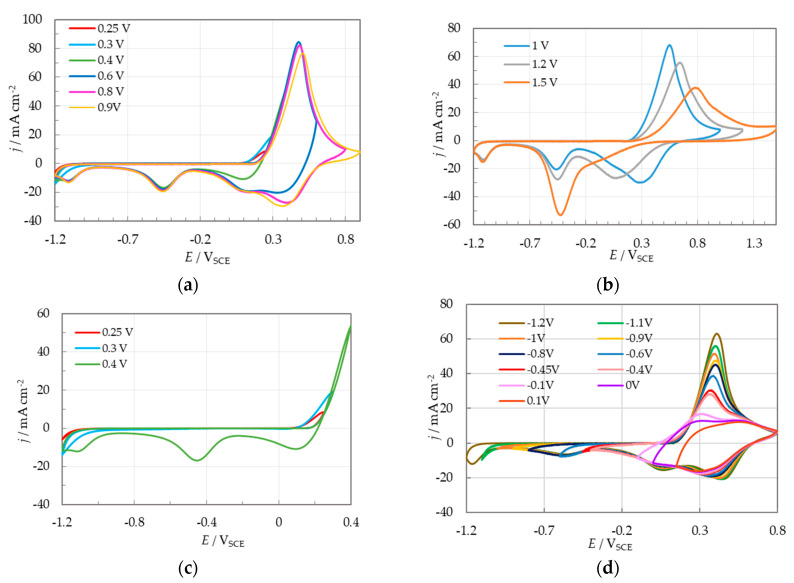
CVs of the Ta|Ta_2_O_5_-PANI|PANI electrode in ANI-free 1 M H_2_SO_4_ traced at *υ* = 50 mV s^−1^ upon successively increasing the upper potential limit: *E*_upper_ in the potential range of 0.25–1.5 V_SCE_ (**a**–**c**), as well as the lower potential limit *E*_lower_ in the potential range of −1.2 V_SCE_–0 (**d**).

To further understand how the anodic behavior of the Ta|Ta_2_O_5_-PANI|PANI electrode is correlated with the multi-cathodic peaks, successive CVs were traced upon varying the upper anodic potential limit *E*_upper_. As seen in Figure 4a, the CVs traced up to *E*_upper_ = 0.9 V_SCE_ retain their main characteristics. On the contrary, significant changes are observed in CVs for which *E*_upper_ > 0.9 V_SCE_ (Figure 4b). To sum up, three characteristic responses can be distinguished in CVs by varying the *E*_upper_.

1. When *E*_upper_ ≤ 0.3 V_SCE_, peaks C3 and C4 disappeared concomitantly with the anodic peaks A1 and A2, as can be clearly seen in Figure 4c.
2. When *E*_upper_ ranged between 0.35 and 0.9 V_SCE_, cathodic peaks C1 and C2 remained almost constant and started to gradually diminish at *E*_upper_ ≤ 0.6 V_SCE_ (Figure 4a). Peaks C3 and C4 remained almost unchanged within the whole potential region.
3. When *E*_upper_ > 0.9 V_SCE_, peaks C1 and C2 gradually merged into a single peak that shifted toward lower potentials. Eventually, at *E*_upper_ = 1.5 V_SCE_, the single cathodic peak originating from the combination of peaks C1 and C2 merged with C3, whereas peak C4 remained unaffected (Figure 4b).

Moreover, the effect of the lower potential limit *E*_lower_ on the redox behavior of the Ta|Ta_2_O_5_-PANI|PANI electrode was investigated in the range between −1.2 V_SCE_ and 0. As can be seen in Figure 4d, the anodic peak decreases as the *E*_lower_ shifts to higher values indicating that both peaks C3 and C4 are correlated with the anodic peak, which is in agreement with the effect observed on the cathodic peaks upon varying the *E*_upper_ (Figure 4a–c).

### 2.3. Effect of the Potential Scan Rate on the Synthesis of PANI

The initiation step of the electrochemical polymerization of ANI on Ta in 1 M H_2_SO_4_ manifests itself through the ANI oxidation peak arising at *E*_p_ = 1.2 V_SCE_ during the first potential cycle, as can be seen in the inset of Figure 1a at *υ* = 50 mV s^−1^. The oxidation of adsorbed monomers, leading to ANI cation radicals, arises out of the passive state of Ta, and the obtained CV is characterized by the crossing of the anodic curve occurring during the reverse backward potential scan. This is indicative of faradaic reactions as, upon reversing the potential scan towards lower values, this process continues to proceed at an enhanced rate and/or with lower overpotential. The origin of this behavior in the present case relates to the coupling of ANI cation radicals, resulting in oligomers that are oxidized at lower potentials [30]. The visual observations of the Ta surface in this region reveal a light-green surface comprising a hybrid surface tantalum oxide-oligomer film.

**Figure 5:**
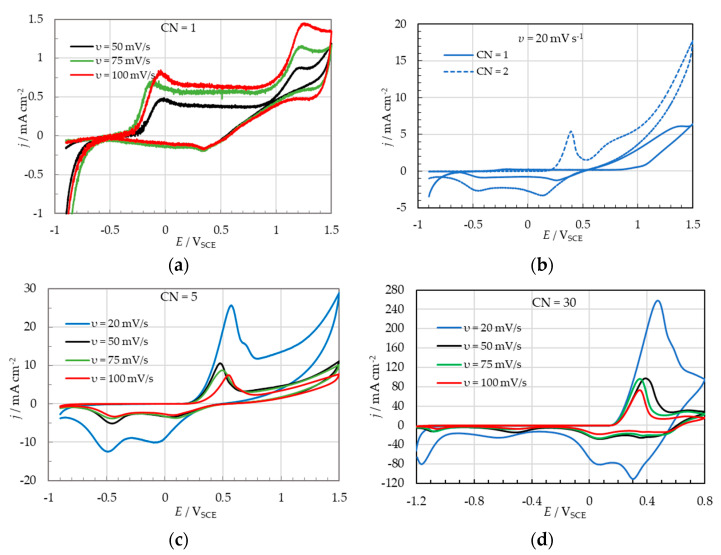
CVs were traced during several stages of the initiation and growth of PANI on the Ta electrode in 1 M H_2_SO_4_ containing 0.2 M ANI to explore the effect of the potential scan rate: (**a**) 1st potential cycle (CN = 1) traced at *υ* = 50, 75 and 100 mV s^−1^; (**b**) 1st and 2nd successive potential cycles (CN = 1, 2) traced at *υ* = 20 mV s^−1^; (**c**) the effect of *υ* on the 5th cycle (CN = 5) within the potential range of −0.9–1.5 V_SCE_; and (**d**) the effect of *υ* on the 30th cycle (CN = 30) within the potential range of −0.9–0.8 V_SCE_.

Figure 5a shows that upon increasing the potential scan rate, though the oxidation peak of ANI at *E*_p_ = 1.2 V_SCE_ does appear, the crossing in the reverse curve of the 1st cycle does not appear, proving that the extent of the follow-up chemical reactions associated with the electron transfer steps of adsorbed dimers/oligomers depends on the time scale of the measurements. As *υ* increases, less time is available for the formation of dimers/oligomers during the scanning of the specific potential region. On the contrary, by decreasing *υ* (Figure 5b), the crossing effect during the 1st potential cycle is more pronounced, and the oxidation current around *E* = 1 V_SCE_ is 10 times higher during the reverse backward potential scan than during the forward one.

Current densities assigned to the redox behavior of the electrodeposited PANI become higher by decreasing *υ* during the polymerization stage within both regions, namely −1.2–1.5 V_SCE_ (Figure 5c) and −1.2–0.8 V_SCE_ (Figure 5d). An increase in the PANI oxidation current observed by decreasing the synthesis potential scan rate was also observed for other substrates, such as AISI 304 stainless steel [17], Ti [22], Pt [34], Ni [35] and Ag [36]. Using *υ* in the range of 10 and 100 mV s^−1^ for the ANI polymerization on Pt shows that the peak current density *j*_p_ linearly decreases due to PANI oxidation, with *υ*^1/2^ indicating a mass-transport control [34]. However, this is not the case for Ta, as the *j*_p_ of peak A1–2 at the end of synthesis (CN = 30) is 3.5 times higher at *υ* = 20 mV s^−1^ compared to that at *υ* = 100 mV s^−1^. This is indicative of the contribution of Ta_2_O_5_ to the polymerization process, perhaps suggesting the chemical oxidation of ANI initiated by the surface oxide, along with the ANI electrochemical oxidation. Therefore, an interfacial accumulation of dimers/oligomers that are electro-oxidized at lower potentials than ANI does result in higher oxidation currents during the reverse backward scan at *υ* = 20 mV s^−1^ (Figure 5b). Further studies of the PANI nucleation/growth mechanism on valve metals are in currently progress.

**Figure 6:**
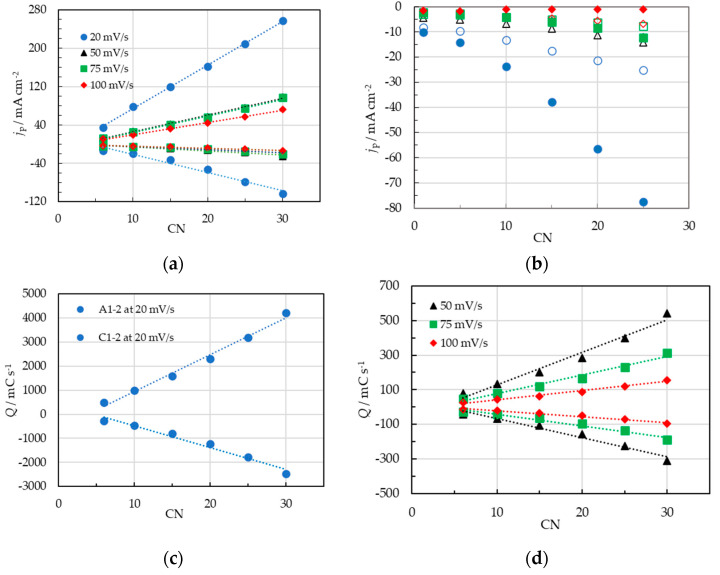
Effect of the cycle number CN on the peak current density *j*_p_ of peaks (**a**) A1–2 and C1–2 and (**b**) C3 (open symbol) and C4 (solid symbol) at various potential scan rates, *υ*, as well as the charge current density *Q* included under peaks A1–2 and C1–2 at (**c**) *υ* = 20 mV s^−1^ and (**d**) *υ* = 50, 75 and 100 mV s^−1^ during the growth of PANI on the Ta electrode in 1 M H_2_SO_4_ containing 0.2 M ANI.

The *j*_p_ for all peaks increases during successive potential cycling in the polymerization solution at all *υ* values used for the PANI electrosynthesis in this work. In particular, the *j*_p_ values for peaks A1–2 and C1–2 linearly increase with CN (Figure 6a). The slope $\left(\frac{\partial j_{p}}{\partial CN}\right)$ of the *j*_p_ = f(CN) plot for peak A1–2 was used as a measure of the ANI electropolymerization rate at various *υ* values (Table 1).

A linear *j*_p_ = f(CN) curve was also observed for peaks C3 and C4 for *υ* > 20 mV s^−1^. At *υ* = 20 mV s^−1^, the *j*_p_ = f(CN) curve deviates from linearity, as, at this scan rate and lower ones, there is an enormous increase in *j*_p_ compared to that observed at higher *υ*. This is better reflected in the charge density *Q* included under the CV corresponding to the peak A1–2, which is almost eight times higher (Figure 6c) than that observed at *υ* = 50 mV s^−1^ (Figure 6d), and hence the corresponding electrodeposition rate $\left(\frac{\partial Q}{\partial CN}\right)$ (Table 1).

The abrupt unexpected enormous increase observed in the current and charge density at lower synthesis scan rates implies a catalytic effect on the ANI polymerization process associated with the hybrid oxide-PANI substrate [22]. This catalytic effect is enhanced at *υ* ≤ 20 mV s^−1^ because a longer time is required to transform the insulating tantalum oxide substrate to the conductive composite tantalum oxide-PANI film. In fact, two different ranges of *υ* are identified for the synthesis of PANI on Ta|Ta_2_O_5_: one at a relatively high *υ* value (*υ*~30–100 mV s^−1^) and another at relatively low *υ* (*υ* < 30 mV s^−1^).

**Figure 7:**
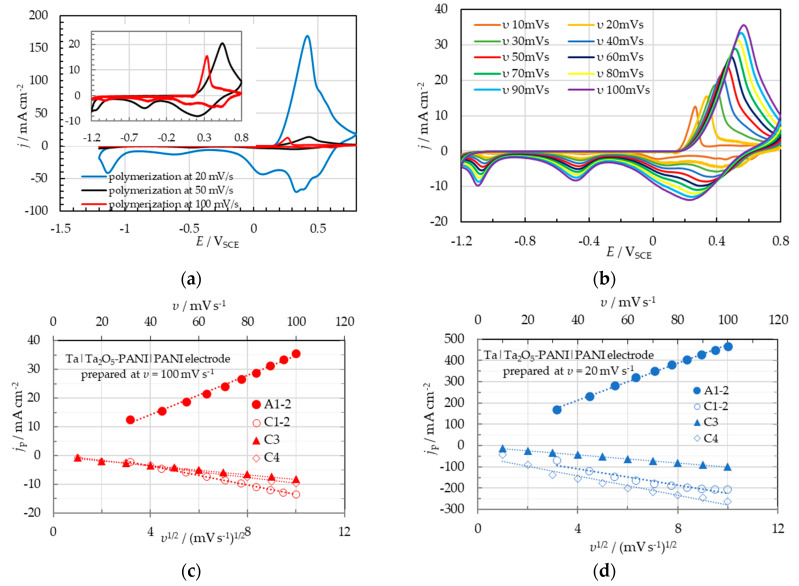
(**a**) The electrochemical response of the Ta|Ta_2_O_5_-PANI|PANI electrode prepared at different potential scan rates in the ANI-free 1 M H_2_SO_4_ solution. CVs were traced within the range of −1.2–0.8 V_SCE_ at *υ* = 10 mV s^−1^. (**b**) The effect of *υ* on the CVs of the Ta|Ta_2_O_5_-PANI|PANI electrode formed at *υ* = 100 mV s^−1^ and (**c**,**d**) the linear dependence of *j*_p_ corresponding to peaks A1–2, C1–2 (*j*_p_ = f(*υ*^1/2^) and C3, C4 (*j*_p_ = f(*υ*) for the synthesis scan rates *υ* = 100 mV s^−1^ (**c**) and *υ* = 20 mV s^−1^ (**d**). The inset in (**a**) shows a magnification of CVs for electrodes prepared at *υ* = 50 mV s^−1^ and *υ* =100 mV s^−1^.

The effect of *υ* on the redox processes was examined for all electrodes prepared at different scan rates, as shown above for *υ* = 50 mV s^−1^ (Figure 3a). Figure 7b depicts examples of CVs obtained at different scan rates for the Ta|Ta_2_O_5_-PANI|PANI electrode prepared at *υ* = 100 mV s^−1^. As for electrodes prepared at *υ* = 50 mV s^−1^, in the case of electrodes prepared at a higher scan rate, namely *υ* = 100 mV s^−1^ (Figure 7c), and a lower one, namely *υ* = 20 mV s^−1^ (Figure 7d), a linear relationship is found between *j*_p_ and *υ*^1/2^ for the peak A1–2 and its counterpart C1–2, as well as between *j*_p_ and *υ* for peaks C3 and C4.

### 2.4. Morphology of PANI Films Electrodeposited on Ta

**Figure 8:**
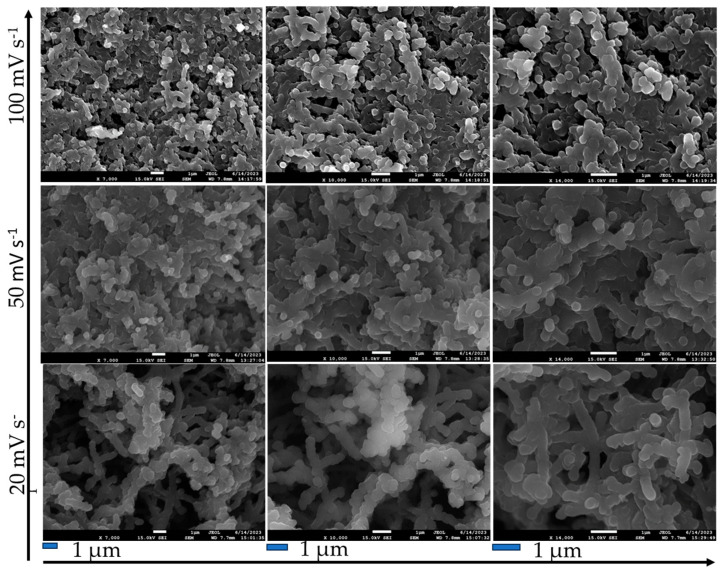
SEM images of the Ta_2_O_5_-PANI|PANI films prepared via cyclic voltammetry at different *υ* values in 1 M H_2_SO_4_ containing 0.2 M ANI following a protocol that comprises 5 cycles within the potential region of −0.9–1.5 V_SCE_ and 25 cycles within the range of −1.2–0.8 V_SCE_.

Several parameters, such as the electrode substrate, the potential scan rate and the potential window, as well as the sulfuric acid and ANI concentrations from which the PANI synthesis was carried out using cyclic voltammetry, were found to affect the morphology, along with other properties of the deposited PANI [2,17,35,36,37,38,39,40]. Examples of SEM images taken for the Ta_2_O_5_-PANI|PANI films deposited on Ta using cyclic voltammetry at *υ* = 20, 50 and 100 mV s^−1^ from 1.0 M H_2_SO_4_ solution containing 0.2 M ANI are illustrated in Figure 8.

**Figure 9:**
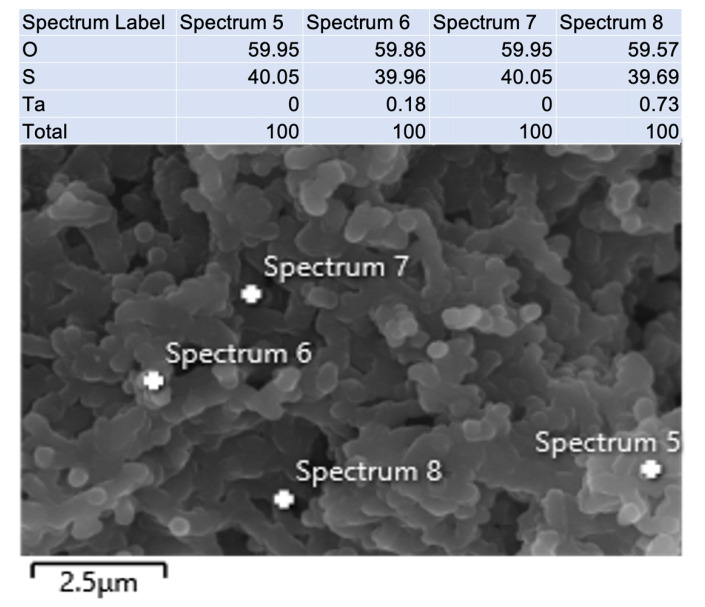
EDS analysis corresponding to the SEM image of the Ta_2_O_5_-PANI|PANI film prepared via cyclic voltammetry at *υ* = 50 mV s^−1^ (for details, see Figure 8).

The morphology observed for the outermost layer of deposits formed at different *υ*, while all other conditions remain the same, is fibrous in nature, with a cluster networking structure. SEM images of Figure 8 reveal that the fibrillar structure is formed by joined nanoparticles in regions where their population is high. The average size of nanoparticles increases by decreasing *υ*, that is, by increasing the polymerization period. Nanoparticles of dimensions ranged between 250 and 400 nm at *υ* = 100 mV s^−1^, 350 and 500 nm at *υ* = 50 mV s^−1^ and 450 and 600 nm at *υ* = 20 mV s^−1^ and agglomerated into interconnected networks until they formed branched network-like nanofibers, the length of which increased by decreasing *υ* and ranged between 1 and 2 μm, 2 and 4 μm and 3 and 8 μm, respectively. As the energy dispersive X-ray spectroscopy (EDS) analysis confirms (Figure 9), this outermost layer of the PANI-modified Ta|Ta_2_O_5_ electrode consists mostly of PANI, whereas Ta is basically absent.

**Figure 10:**
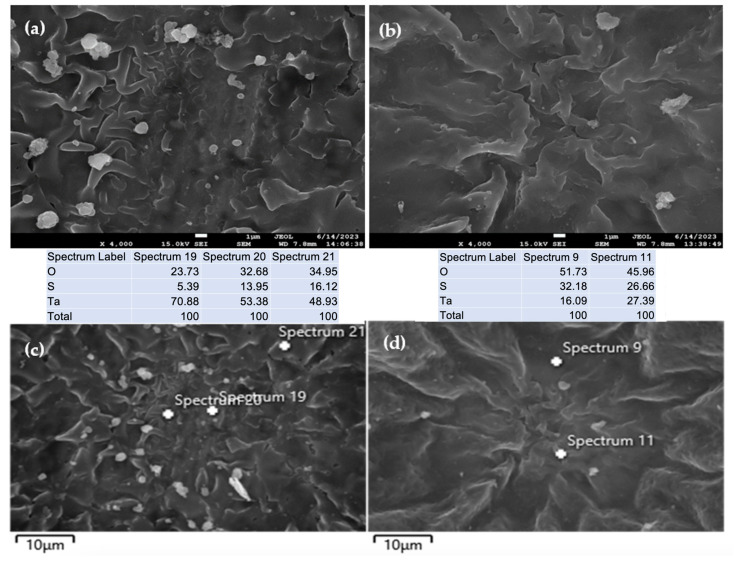
SEM images and corresponding EDS analysis of the Ta_2_O_5_-PANI composite films prepared via cyclic voltammetry at *υ* = 50 mV s^−1^ (for more details, see Figure 8) after removing the outermost PANI layer at the early (**a**,**c**) and late (**b**,**d**) stages of the polymerization process.

The morphology of the outermost PANI is closely associated with the co-deposition of the tantalum oxide in the stage of nucleation and growth of oligomer/polymer, resulting in the growth of the inner Ta_2_O_5_-PANI composite layer. The conductivity of the latter layer is enhanced compared to that of Ta_2_O_5_, while SEM images in Figure 10a,b reveal that it is characterized by a more compact structure than that of the outer layer.

EDS analysis of the inner layer (spectra in Figure 10c,d) in SEM images taken at different polymerization periods indicates, in contrast to the outermost PANI layer (Figure 9), the presence of Ta that originates from the tantalum oxide in the compact, homogeneous and well-adherent Ta_2_O_5_-PANI composite film and the substrate.

### 2.5. Galvanostatic Charge/Discharge Curves

**Figure 11:**
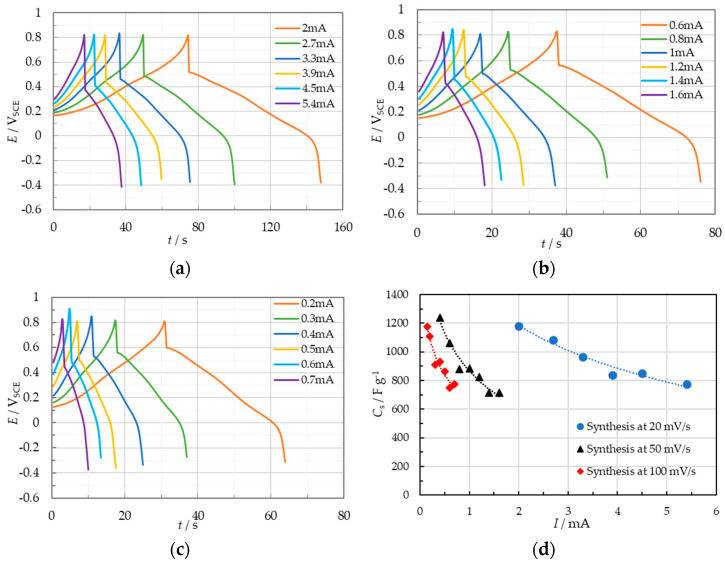
GCD curves obtained at different charge–discharge current values in 1 M H_2_SO_4_ for the Ta|Ta_2_O_5_-PANI|PANI electrodes prepared at different *υ* values: (**a**) *υ* = 20 mV s^−1^, with GCD curves in the range of 12.1–46.7 A g^−1^; (**b**) *υ* = 50 mV s^−1^, with GCD curves in the range of 18.3–73.4 A g^−1^; and (**c**) *υ* = 100 mV s^−1^, with GCD curves in the range of 23.8–110.9 A g^−1^. (**d**) The effect of the charge-discharge current on the specific capacitance, *C_s_*.

To examine the electrochemical performance of the Ta|Ta_2_O_5_-PANI|PANI as an electrode for supercapacitors, galvanostatic charge-discharge (GCD) E-t curves were recorded at different current values in the potential range of −0.35–0.8 V_SCE_ in a 1.0 M H_2_SO_4_ solution (Figure 11). Different ranges of current densities were used to record GCD curves for electrodes prepared at different υ values. The GCD E-t curves exhibit an asymmetric shape during anodic charging and cathodic discharging processes, which reflects the redox behavior (Figure 5 and Figure 7) of the Ta|Ta_2_O_5_-PANI|PANI electrodes, indicating the pseudocapacitive charge storage occurring in them. Due to the non-linear GCD curves, the Equation (3) (in Section 4) was used for the estimation of the specific capacitance *C_s_*. In all electrodes, there is a decrease in the charging and discharging time upon increasing the applied current (Figure 11a–c). In general, relatively high *C_s_* values were obtained for all prepared electrodes (Figure 11d). It seems that by decreasing the synthesis potential scan rate, the *C_s_* increases, whereas it decreases upon increasing the charge-discharge current.

The higher values of *C_s_* estimated for the electrodes prepared at lower *υ* values agree with the more open structure observed in SEM images, as nanospheres agglomerate to nanofibers upon decreasing *υ* (Figure 8). However, within the range of the charge-discharge current values used in this study, the obtained *C_s_* values for Ta_2_O_5_-PANI|PANI films are comparable to the relatively moderate-to-high values reported in the literature for various PANI-based electrodes [10]. Moreover, the capacitance retention after 100 repeated cycles of the charging–discharging process between −0.35 and 0.8 V_SCE_ was ~80%. Further studies investigating the effect of the PANI thickness and nucleation/growth mechanisms of PANI on the capacitive properties of the Ta_2_O_5_-PANI|PANI are currently in progress.

## 3. Discussion

The presented results show that cyclic voltammetry is an effective technique for modifying the low-conductivity n-type Ta_2_O_5_ formed on the Ta surface and transforming it into the conductive Ta_2_O_5_-PANI composite layer on which the electrodeposition of PANI occurs at relatively high rates depending on *υ* value used for the polymerization (Table 1). A decrease in the polarization resistance of about two orders of magnitude was observed for the Ta_2_O_5_-PANI layer compared to the pure Ta_2_O_5_ layer [41] using electrochemical impedance spectroscopy, which is in agreement with similar previous studies on Nb [24]. In this way, the application of tantalum oxide used in biosensors [42,43] for resistive switching memories [44] or as a self-supported material in lithium batteries [45], to mention only two examples, can be extended or improved.

**Figure 12:**
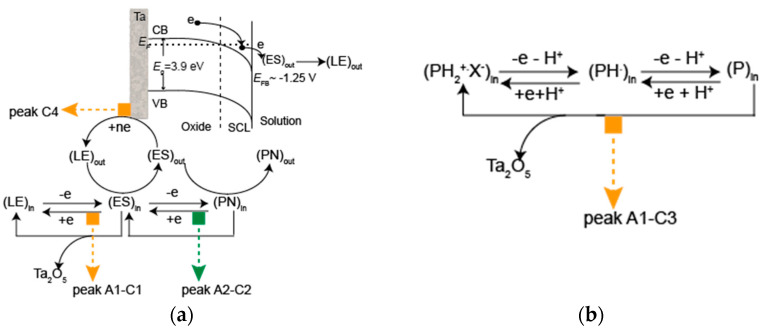
Schematic illustration of the redox processes assigned to the anodic peaks A1 and A2 and cathodic peaks C1, C2, C3 and C4 appeared in the cyclic voltammograms of the Τa|Τa_2_O_5_-PANI|PANI electrodes in 1 M H_2_SO_4_. (**a**) The two-stage ES-to-LE transition corresponding to a direct transfer of electrons from the substrate to emeraldine salt (ES)_in_ at the inner Ta_2_O_5_-PANI layer and the reduction of the (ES)_out_ in the vicinity of the flat-band potential *E*_FB_ of the tantalum oxide (*E*_FB_~−1.25 V_SCE_) via an electron accumulation layer formed at the surface of the composite layer. (**b**) The two-stage redox processes assigned to phenazine derivatives that are presumably formed during the initiation stage of the ANI polymerization on the hydrophobic Ta_2_O_5_ surface.

The variation in *υ* is an important parameter of the cyclic voltammetric technique, allowing the control of the structure and morphology of deposits. Using *υ* < 30 mV, s^−1^ greatly enhances the growth of PANI. In fact, at these relatively low *υ* values, the electropolymerization time and the amount of material deposited abnormally increase due to the progressive decrease in the resistance of the inner Ta_2_O_5_-PANI composite film. It is possible that a synergistic chemical oxidation of monomer/oligomers/polymer caused by the tantalum oxide occurs. Moreover, as the electropolymerization time increases, single PANI is deposited on top of the Ta_2_O_5_-PANI composite film, which gradually starts to agglomerate via growth on the previously formed polymer, increasing the thickness and changing the morphology from spherical micro-nanoparticles to micro-nanofibers. Controlling the film morphology at the micro-nanoscale affects several properties of the deposited PANI-based layers for a variety of applications, such as supercapacitors for energy storage, as shown by the preliminary results presented in Figure 12.

In general, the electrodeposition of PANI and other CPs on metal substrates involves complex interfacial processes, such as mass transport, heterogeneous and homogeneous electron transfer, the formation of oligomers, polymerization, subsequent deposition on the metal surface, the solubility of some species and doping comprising ion/solvent exchange for charge balance [46]. The PANI electrodeposition on valve metals seems to involve additional interfacial processes, including the oxide co-deposition, a solid-state electron transfer and other transformations of adsorbed oligomers, leading to the formation of the Ta_2_O_5_-PANI composite layer and the growth of PANI on top of the inner layer. This different interfacial mechanism, in the case of Ta, manifests itself through two additional cathodic peaks, namely C3 and C4, in CVs of the prepared Ta|Ta_2_O_5_-PANI|PANI electrodes (Figure 1, Figure 2, Figure 3 and Figure 4).

Cyclic voltammetry allows the monitoring of the redox behavior of the surface-bound material during the co-deposition of both tantalum oxide and PANI, as well as the deposition of single PANI on top of it. The prepared Ta|Ta_2_O_5_-PANI|PANI electrodes are stable in the ANI-free 1 M H_2_SO_4_ and retain the redox behavior observed during polymerization. Their electrochemical response under either the presence or absence of ANI in sulfuric acid solutions differs from that frequently reported in the literature for PANI-modified electrodes. As mentioned above, besides the redox peaks corresponding to transitions between different states of PANI, namely the LE⟶ES and ES⟶PN transitions, CVs disclose additional cathodic peaks, designated as C3 and C4 in this work (Figure 1). The main characteristics of peaks C3 and C4 are as follows: (i) both peaks remain almost stable during the potential cycles (Figure 2); (ii) their *j*_p_ values linearly vary with the potential scan rate (Figure 3), indicating a surface-bound species; (iii) the total anodic charge is equal to the total cathodic one, i.e., *Q*c_tot_ = *Q*_C1–2_ + *Q*_C3_ + *Q*_C4_ (Figure 2b); (iv) both peaks are related to a part of the anodic peak A1 since they disappear once using *E*_upper_ < 0.25 V_SCE_, at which the peak C1, as the counterpart of A1, disappears as well (Figure 4a–c); and (v) asymmetry in the redox A1–2/C1–2 pair, in which the anodic peak A1–2 gradually decreases by decreasing the *E*_lower_ to reach a value of *j*_p,A_ that is almost equal to the cathodic one, namely *j*_p,C_ (Figure 4d), occurs, indicating that a part of the anodic peak has its counterpart either in peak C3 and/or C4.

Similar behavior was also observed in the case of Ti [21,23] and Nb [24], where only peak C3, however, appeared. Based on the possible polymerization paths of ANI investigated in detail under various experimental conditions in the literature [2], as well as the fact that the additional reduction peak so far only appears when these specific valve metals were used as substrates, the “model of phenazine nucleates” could perhaps explain the appearance of peak C3, as was previously postulated for Ti [21] and Nb [24].

According to this model, it is suggested that the hydrophobic Ta_2_O_5_ surface facilitates the initiation of aniline trimers containing a phenazine moiety [2], which is also a hydrophobic species with low solubility in water. Though the exact chemical structures of these phenazine-containing moieties are not fully understood, there is evidence that they have flat molecular structures and hence can facilitate stacking through π-π interactions acting as nucleates. PANI developed from this adsorbed oligomer that nucleates on the flat Ta|Ta_2_O_5_ surface is expected to be uniform or globular in morphology at the initiation stage of polymerization, as SEM images have shown (Figure 10). The hydrophobic phenazine-containing part is fixed toward the hydrophobic Ta_2_O_5_ surface, and the growth of the polymer continues from the hydrophilic protonated part.

Phenazine (P) moiety in the developed polymer is expected to be oxidized at potentials where the LE⟶ES transition approximately occurs, albeit at slightly lower potentials [31]. Though the decoupling of the anodic peak A1–2 is not clearly observed, the corresponding cathodic behavior exhibits distinct peaks, namely peak C3, assigned to the reduction of the oxidized form of phenazine derivative, (PH_2_^+.^X^−^) (Figure 12b), along with peaks C1 and C2, assigned to the (PN)_in_⟶(ES)_in_ and (ES)_in_⟶(LE)_in_ reductive transitions (Figure 12a) of the inner composite layer of the surface-bound material.

As a direct electron transfer from the Ta substrate to the PANI outer layer may be prevented due to the semiconducting character of the oxide layer [47], it is plausible to suggest that the electroreduction would be facilitated close to the flat-band potential *E*_FB_~−1.25 V_SCE_ of Ta_2_O_5_, where peak C4 is located due to the bending of the conduction band (CB) and the valence band (VB) that lead to an electron accumulation layer across the oxide [48], as shown in Figure 12a. Indeed, the results of this study, combined with those reported for TiO_2_ and Nb_2_O_5_, support such a correlation, as the *E*_FB_ for both metal oxides being around −0.35 V_SCE_ differs remarkably from that of Ta_2_O_5_. This difference implies that the presence of peak C3 in the case of Ti and Nb substrates originates from both the phenazine moiety and the semiconducting properties of Nb_2_O_5_ and TiO_2_. In the case of the Ta substrate, distinct peaks appear for these processes, namely peak C3 for phenazine derivatives and peak C4 located at ~−1.25 V_SCE_, adjacent to the *E*_FB_ of Ta_2_O_5_ for the reduction of (LE)_out_ (Figure 12a). The observation of the cathodic peak C4 is discussed for the first time in this study, and additional research is underway to provide further evidence supporting the co-deposition mechanism of oxide and PANI or other CPs on valve metal substrates.

## 4. Materials and Methods

**Scheme 1:**
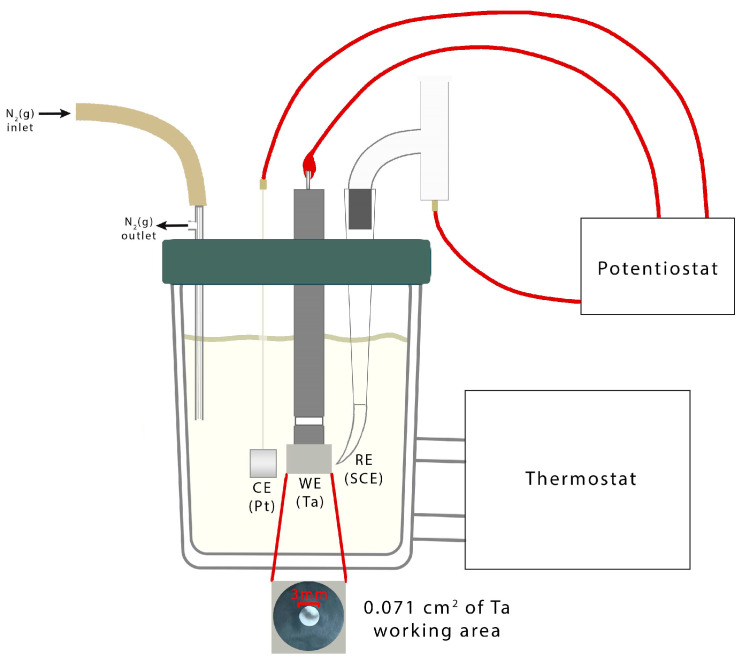
Schematic of the three-electrode electrochemical cell used for the electrochemical measurements.

The electrochemical polymerization of PANI on the Ta electrode was conducted from a 1 M H_2_SO_4_ (Merck, proanalysis 96%, *w*/*w*) solution containing 0.2 M ANI (Sigma Aldrich, ACS reagent, ≥99.5%) in a conventional three-electrode electrochemical cell, as shown in Scheme 1. The working electrode (WE) was the cross section of a Ta rod (99.99% in purity from Goodfellow) with a diameter equal to 3 mm embedded in a 1 cm diameter PTFE cylinder (surface area = 0.071 cm^2^). A Pt sheet (2.5 cm^2^ in surface area) was served as the counter electrode (CE). The reference electrode (RE) was a saturated (in KCl solution) calomel electrode (SCE). The electrochemical cell was kept at 298 K using a thermostat, while N_2_ of high purity was utilized for the de-oxygenation of the solution. N_2_ was passed at a slow flow rate above the solution during the measurement stage. The electrochemical measurements were carried out using a VoltaLab 40 electrochemical system accompanied by the VoltaMaster 4 software from Radiometer Analytical.

Twice-distilled water was used for the preparation of the solutions for both the polymerization and characterization of PANI-modified Ta electrodes. Prior to the electrochemical polymerization of ANI, the Ta electrode was first mechanically polished with wet abrasive papers of different grit sizes (600, 800, 1000, 1200 and superfine) and then cleaned in an ultrasonic bath containing twice-distilled water. The open-circuit potential was measured after the immersion of the Ta electrode in the polymerization solution and prior to the beginning of the polymerization process to ensure reproducible surface conditions, as the formation of a thin native oxide on the Ta surface was unavoidable. After the PANI electrodeposition, the obtained electrodes, designated as Ta|Ta_2_O_5_-PANI|PANI, were washed using distilled water and transferred into an ANI-free 1 M H_2_SO_4_ solution for the characterization of their redox properties, stability and capacitive properties.

The electrochemical oxidation of ANI and the subsequent polymerization and simultaneous deposition of PANI films on the prepared Ta|Ta_2_O_5_ electrodes were carried out using cyclic voltammetry. A protocol comprising 5 successive potential cycles at a potential scan rate of *υ* = 50 mV s^−1^ within the potential region of −0.5–1.5 V_SCE_, followed by 25 cycles between −0.5 and 0.8 V_SCE_, was mostly utilized, except for measurements involved the investigation of the effect of *υ* on the electrodeposited PANI. The electrochemical response and the stability of the prepared Ta|Ta_2_O_5_-PANI|PANI electrodes were examined in ANI-free 1 M H_2_SO_4_ solution at *υ* = 50 mV s^−1^. Successive potential cycling up to 50 cycles in the latter solution to reach an almost steady-state CV was required before studying the effect of *υ* on the peak current densities of the PANI redox response.

The *E*_FB_ of the Ta_2_O_5_ formed in 1 M H_2_SO_4_ solution at 1.5 V_SCE_ was estimated via the Mott–Schottky analysis [49] of electrochemical impedance spectra obtained by applying an AC potential of 10 mV at a constant frequency of *f* = 5 KHz.

GCD *E*-*t* curves at different applied current values, Ι were traced within a certain potential window Δ*Ε* to evaluate the specific capacitance *C_s_* of the Ta|Ta_2_O_5_-PANI|PANI electrodes and identify a first sign of their competence as energy materials. The specific capacitance can be estimated using the following equation:

(2) $$C_{s}=\frac{I\cdot \Delta t}{m\cdot \Delta Ε}$$

where *C_s_* is the specific capacitance in F g^−1^, *Ι* is the applied current in A, Δ*E* is the operational potential window for the charge–discharge process in V, Δ*t* is the discharge time in s and *m* is the mass of the active material in g estimated using Faraday’s law. The charge was evaluated via the integration of the polymerization oxidation peaks.

However, due to the asymmetric non-linear profiles of the GCD curves, observed in the case of pseudocapacitive materials [50], the following equation was used for the estimation of *C_s_*:

(3) $$C_{s}=\frac{2I}{m\cdot {\left(\Delta E\right)}^{2}}\int_{E-}^{E+}(\Delta E)dt$$

where *E*+ and *E*− are the maximum and minimum values of the operational potential window Δ*E*, $\int_{E-}^{E+}(\Delta E)dt$ is the current integral area and all other symbols are as defined above.

The morphology of the PANI was examined using Field-emission SEM, JEOL JSM-7610F Plus supported by an Oxford AZTEC ENERGY ADVANCED X-act energy dispersive X-ray spectroscopy system. SEM images were acquired in the low-vacuum mode, with operating conditions of a 20 kV accelerating voltage, a 1 nA probe current and a working distance of 10 mm. Energy Dispersive Spectra were also acquired to evaluate the local composition. The maximum information depth for the EDS analysis was 2 μm, whereas a SEM spot size of 1 μm^2^ was used. The software Gwyddion 2.63, developed by the Department of Nanometrology, Czech Metrology Institute, was used for the calculation of the morphological parameters in SEM images.

## 5. Conclusions

Bi-layer well-adherent Ta_2_O_5_-PANI|PANI films were successfully deposited at adequately high rates on Ta from 1 M H_2_SO_4_ aqueous solution containing 0.2 M ANI using cyclic voltammetry. Tuning the potential scan rate and other electrochemical parameters allowed the control of electrochemical properties and other features of the formed surface film, including its thickness, structure and morphology. The main results of this study are summarized as follows:

- The redox behavior of PANI exhibits two additional cathodic peaks, apart from the expected redox behavior comprising transitions between different oxidation states. This behavior was not previously observed for the Ta electrode. It is suggested that the cathodic peak close to −1.2 V_SCE_ is related to the n-type semiconducting properties of the Ta_2_O_5_, as it appears close to its *E*_FB_, and the other one, located at less negative potentials, is associated with the reduction of phenazine species formed during the initiation step of polymerization.
- Polymerization occurs simultaneously with the oxide layer, and PANI seems to develop from the adsorbed oligomer nucleates comprising hydrophobic phenazine-containing species fixed toward the hydrophobic Ta_2_O_5_ surface. The growth of the PANI continues, presumably from the hydrophilic protonated part of nucleates with enhanced polymerization rates due to the gradual increase in the conductivity in the inner Ta_2_O_5_-PANI composite layer, affecting the PANI morphology.
- The morphology of PANI films depends on the synthesis potential scan rate. It comprises nano- or microspheres that can be joined, leading to nano- or microrods/fibers with a cluster networking structure.
- Preliminary galvanostatic charge-discharge experiments indicate that the prepared Ta|Ta_2_O_5_-PANI|PANI electrodes display sufficient capacitive properties, being dependent on the potential scan rate used for the synthesis of PANI. The obtained specific capacitance varies between ~700 and ~1200 F g^−1^ at current density values ranging from ~75 to ~2 mA cm^−2^ in an operating potential window of −0.35–0.8 V_SCE_. Certainly, further studies are required to characterize the capacitive responses and cycling stability values of these electrodes.
- Finally, it should be mentioned that the results of this study can give valuable leads to control the formation of PANI films with a desired morphology and properties on other valve metals, extending their application range.

## Figures and Tables

**Table 1:** The effect of the potential scan rate *υ* on the ANI electropolymerization rate $\left(\frac{\partial j_{p}}{\partial CN}\right)$ or the PANI electrodeposition rate $\left(\frac{\partial Q}{\partial CN}\right).$

*υ* (mV s^−1^) for PANI Synthesis	$\left(\frac{\partial j_{p}}{\partial CN}\right)$ ^1^	$\left(\frac{\partial Q}{\partial CN}\right)$ ^1^
20	9.11	152.54
50	3.48	18.78 ^1^
75	3.46	10.82
100	2.56	5.35

^1^ The polymerization and deposition rates were evaluated by considering the anodic peak A1–2.

## Data Availability

Data is contained within the article.
